# A novel frameshift mutation in the *PITX2* gene in a family with Axenfeld-Rieger syndrome using targeted exome sequencing

**DOI:** 10.1186/s12881-019-0840-9

**Published:** 2019-06-11

**Authors:** Lusi Zhang, Yingqian Peng, Pingbo Ouyang, Youling Liang, Huilan Zeng, Nuo Wang, Xuanchu Duan, Jingming Shi

**Affiliations:** 1 Department of Ophthalmology, The Second Xiangya Hospital, Central South University, 139 Renmin Middle Road, Changsha, 410011 Hunan People’s Republic of China; 2 Hunan Clinical Research Center of Ophthalmic Disease, Changsha, Hunan People’s Republic of China

**Keywords:** Axenfeld-Rieger syndrome, *PITX2*, Targeted exome sequencing, Frameshift variation

## Abstract

**Background:**

Axenfeld-Rieger syndrome (ARS) is an autosomal dominant genetic disorder that is characterized by specific abnormalities of the anterior segment of the eye. Heterozygous mutations in two developmental transcription factor genes *PITX2* and *FOXC1* have been identified within ARS patients, accounting for 40 to 70% of cases. Our purpose is to describe clinical and genetic findings in a Chinese family with ARS.

**Methods:**

An ARS family with three affected members was recruited. The patients underwent a series of complete ophthalmologic examinations, general physical examination and dental radiography. DNA samples of proband II-1 were used for targeted exome sequencing of the *FOXC1* and *PITX2* genes. Sanger sequencing was used to validate the variation in *PITX2*. Quantitative real-time PCR was carried out to detect the expression of *PITX2* in patients and normal controls.

**Results:**

All affected members showed iris atrophy, corectopia, shallow anterior chamber, complete or partial angle closure, and advanced glaucoma. In addition, they revealed systemic anomalies, including microdontia, hypodontia, and redundant periumbilical skin. A novel heterozygous frameshift variation, c.515delA, in *PITX2* was found in the proband, which might lead to a truncated PITX2 protein (p.Gln172ArgfsX36). Sanger sequencing validated that the variation completely cosegregated with the ARS phenotype among this family, but was absent in 100 unrelated controls. Quantitative real-time PCR analysis revealed that the mRNA expression of *PITX2* was significantly decreased in patients compared with that in unrelated normal controls.

**Conclusions:**

*PITX2* c.515delA (p.Gln172ArgfsX36) was the genetic etiology of our pedigree. The mutation led to decreased *PITX2* gene expression and a truncated mRNA transcript.

## Background

Axenfeld-Rieger syndrome (ARS) is an autosomal-dominant genetic disorder that is characterized by specific abnormalities of the anterior segment of the eye [1]. The diagnosis of ARS refers to a serious of ocular phenotypes, including anomalies of the anterior chamber angle and aqueous drainage structures, iris hypoplasia, corectopia (eccentric pupil), polycoria (iris tears), and iridocorneal tissue adhesions traversing the anterior chamber [2].Because of these changes in the trabecular meshwork and iris, patients are at high risk for raised intraocular pressure, and an increased incidence of glaucoma affecting approximately 50% of cases has been reported [3]. Systemic disorders can also occur with these eye anomalies, which include dental hypoplasia, facial dysmorphism, abnormality of the cardiovascular outflow tract, failure of periumbilical skin involution, and maxillary hypoplasia [4, 5]. However, these systemic disorders often reveal incomplete penetrance and variable expressivity.

To date, heterozygous mutations in two developmental transcription factor genes *PITX2* and *FOXC1* have been identified within ARS patients, accounting for 40 to 70% of cases [6, 7]. Additionally, two other loci on 13q14 and 16q24 have been suggested. However, the underlying genes at these loci have not been identified [8, 9]. For significant clinical and genetic heterogeneity, affected patients exhibit various ocular and systemic abnormities, which often complicates diagnosis. In this sense, genetic analysis combined with clinical diagnosis is becoming a promising way to clarify diagnostic classification.

In recent years, exome sequencing has provided a powerful tool to identify causative genes in Mendelian and complex disorders [10, 11]. In the present study we aimed to identify the genetic etiology underlying a Chinese pedigree with ARS through targeted exome sequencing.

## Methods

### Subjects and clinical evaluation

**Fig. 1:**
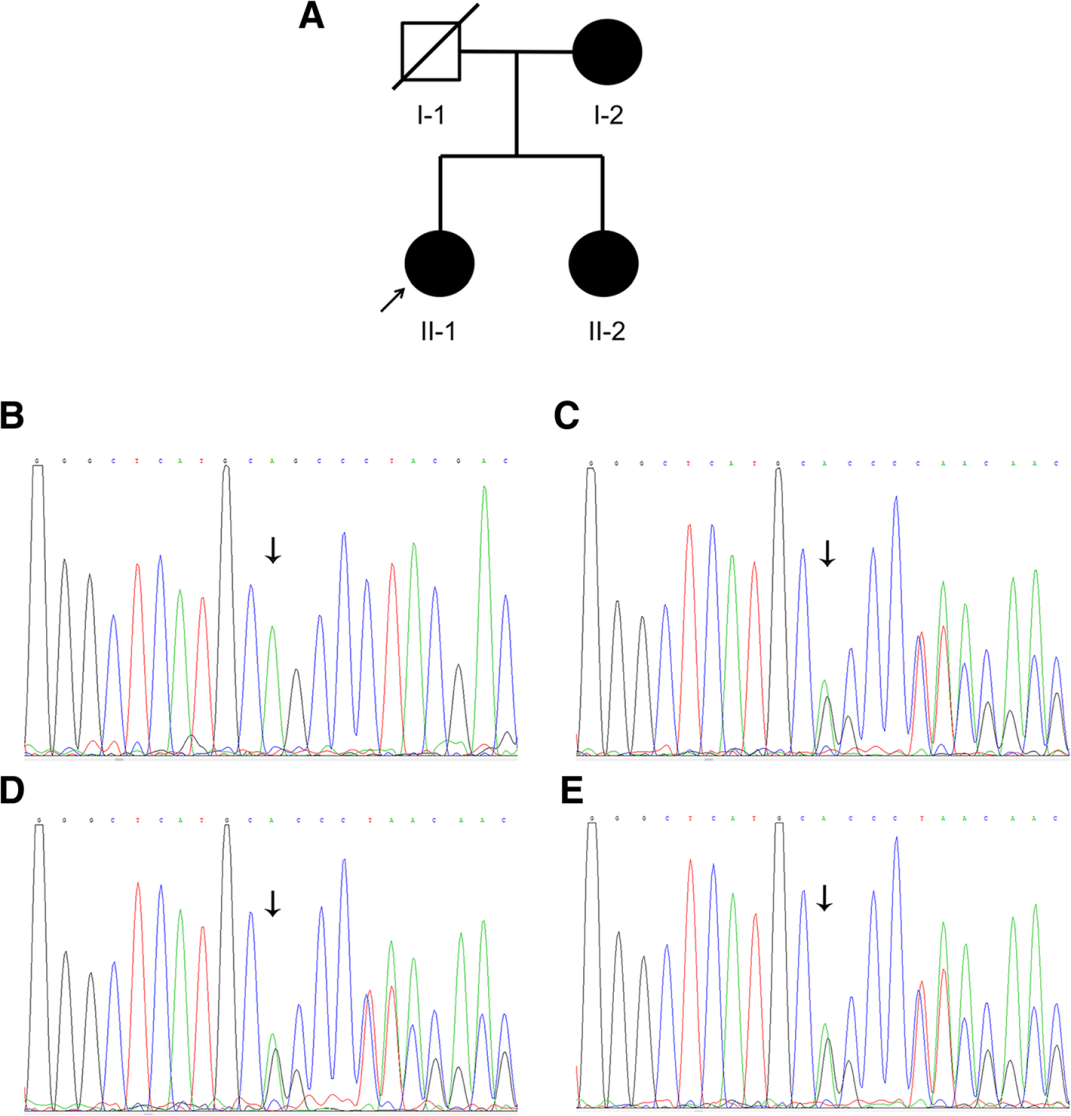
Pedigree and sequence analysis of the PITX2 gene in a Chinese family with Axenfeld-Rieger syndrome. **a** Roman numerals refer to generations, and individuals within a generation are numbered from left to right. Proband II-1 is noted with an arrow; the filled symbol refer to the patients; and the slash refers to the dead individual. **b**-**e**, the DNA sequence of the *CHM* c.280delA mutation in the normal control (**b**) and the patients (**c**, I-2; **d**, II-1; and **e**, II-2). The arrow indicates the mutated base

A Chinese Han family with three affected individuals was included in the study (Fig. 1a). A total of 100 normal controls were recruited mainly from volunteers. The study adhered to the principles of the Declaration of Helsinki, and the clinic protocols were approved by the Second Xiangya Hospital Institutional Review Board. After obtaining written informed consent from patients and the parent, peripheral blood samples were obtained from the family members and the normal volunteers, and DNA was extracted using a standard phenol chloroform method.

All affected members underwent standard ophthalmic examinations including evaluation of best-corrected visual acuity (BCVA), Goldman applanation tonometry, slit-lamp biomicroscopic examination and fundus examination via direct and indirect ophthalmoscopy. Other clinic characterizations of affected individuals included funduscopy (nonmydriatic retinal camera, TRC-NW300, Topcan, Itabashi-ku, Tokyo, Japan), anterior segment photography (SL-D4, Topcan, Itabashi-ku, Tokyo, Japan) and ultrasound biomicroscopy (UBM) as well as assessment of visual field defects performed with a Humphrey vsual field analyzer (Carl Zeiss Humphrey Systems, Dublin, CA, USA). All participants were examined by a general physician and a dentist for the presence or absence of systemic and dental abnormalities. All patients also underwent general physical examination and dental panoramic radiography. The diagnosis of ARS was based upon medical history, clinical features and heredity of the disease.

### Targeted exome sequencing

Genomic DNA was extracted according to the standard phenol–chloroform method. A DNA sample of proband II-1 was used for targeted exome sequencing of the *FOXC1* and *PITX2* genes. Library construction was performed by the NimbleGen SeqCap EZ System (Roche NimbleGen, Madison, Wisconsin, USA) according to the manufacturer’s instructions. 90 cycle paired-end sequencing was performed on Illumina HiSeq2500 Analyzers (Illumina, San Diego, California, USA) following the manufacturer’s instructions. Illumina Pipeline software (version 1.3.4) was used to perform base-calling and to calculate the quality values for every base.

Reads were aligned to the human reference genome National Center for Biotechnology Information (NCBI) GRCh37 using Burrows-Wheeler Aligner (BWA). Single nucleotide variations (SNVs) and insertions and deletions (indels) identification was performed by SOAPsnp and samtools. SNVs and indels with read depth ≥ 8× and quality ≥30 were reserved for subsequent analysis. Based on the dbSNP database and the 1000 genomes annotation, the polymorphic SNVs were excluded. SNVs and indels affecting coding sequence were annotated using Annotate Variation (ANNOVAR) software.

### Sanger sequencing of implicated genes

PCR amplification and Sanger sequencing of the amplicons were used to validate the pathogenic mutation in *PITX2* (NM_000325) identified via exome sequencing. The primer sequences are shown as follows: forward primer, 5′-cactgtggcatctgtttgct-3′, and reverse primer, 5′-ACGGGCTACTCAGGTTGTTC-3′. PCR was performed in a 10-μl reaction mixture using 2 × TaKaRa Taq™ HS Perfect Mix (Takara Biotechnology, Dalian, China). The amplification conditions consisted of an initial denaturation step at 94 °C for 30 s, followed by 33 cycles of denaturation at 94 °C for 5 s, annealing at 60 °C for 20 s, and extension at 72 °C for 20 s. Final extension was performed at 72 °C for 7 min.

### RNA expression analysis by real-time quantitative PCR

Total RNA was extracted from peripheral blood lymphocytes of three patients as well as three unrelated normal controls using TRIzol reagent (Life Technologies, NY, USA). cDNA was synthesized from 0.1 μg of total RNA using the RevertAid First Strand cDNA Synthesis Kit (Thermo Scientific, Inc., Waltham, MA, USA) and oligo (dT) primers. Quantitative real-time PCR was carried out with an Applied Biosystems®StepOne™ Plus Real-Time PCR System (Thermo Scientific, Inc., Waltham, MA, USA) using Maxima SYBR Green qPCR Master Mixes (Thermo Scientific, Inc., Waltham, MA, USA). Data were normalized to β-actin and analyzed by the comparative CT method. Specific primers for each gene were as follows: forward primer *PITX2*–1-F, 5′-TACCTGTCCCTGTCACTCTTGA-3′, and reverse primer *PITX2*–1-R, 5′-AAGAACCCCTCCAATAAGGAAA-3′ for *PITX2* (before c.515); forward primer *PITX2*–2-F, 5′-CCTTACATCCGCCTCCCTAT-3′ and reverse primer *PITX2*–2-R, 5′-GTGGGGAAAACATGCTCTGT-3′ for *PITX2* (after c.515); forward primer, 5′-CACGATGGAGGGGCCGGACTCATC − 3′, and reverse primer, 5′-TAAAGACCTCTATGCCAACACAGT-3′ for β-actin. Statistical analyses were performed using Prism 5 software (GraphPad Software, Inc., La Jolla, CA, USA). Two-tailed Student’s t test was used to determine the significance of differences between two groups. The data are presented as the mean ± SEM.

## Results

### Clinical characterization

This family had three affected members: the proband, her mother, and her younger sister (Fig. 1a). Table 1 and Table 2 summarize the clinical characteristics of the ocular and systemic features of the three patients in this family. Proband II-1 was a 19-year-old female referred to our outpatient clinics because of uncontrolled intraocular pressure (IOP) in both eyes under medical therapy and was diagnosed with iridocorneal endothelial (ICE) syndrome by other hospitals. Case 2 (I-2), a 42-year-old woman, was the mother of the proband who had a trabeculectomy in her left eye 14 years ago because of uncontrolled high IOP (≥ 60 mmHg). Her right eye was blind since childhood, and she never received treatment. Ocular examinations showed adherent leukoma of her right eye. Case 3 (II-2) was the 14-year-old sister of the proband who was referred to our clinic because of low visual acuity in both eyes since childhood.

**Table 1:** Summary of clinical features of affected family members with Axenfeld-Rieger syndrome

Affected family members		I-2	II-1	II-2
Currente age (years old)		42	20	14
Onset age (years old)		28	18	11
Gender		F	F	F
Clinical Features				
Eye	Iris dysplasia (goniodysgenesis)	+	+	+
	Iris hypoplasia	+	+	+
	Pupil deformation	+	+	+
	Secondary Glaucoma	+	+	+
	Polycoria	+	+	+
	Corectopia (displaced pupils)	+	+	+
	Shallow anterior chamber	+	+	+
Nose	Broad nasal bridge	+/−	+	+
Teeth	Microdontia	+	+	+
Abdomen	Umbilical defect (redundant periumbilical skin)	+	+	+

F-Female

**Table 2:** Ocular examinations of affected family members with Axenfeld-Rieger Syndrome

Ocular examinations	I-2		II-1		II-2	
	OD	OS	OD	OS	OD	OS
BCVA	NLP	20/200	20/20	20/33	20/50	20/33
IOP (Goldman tonometry, mmHg)	N/A	21^a^	25	40	34	34
CCT (μm)	N/A	618	562	579	560	564
C/D	N/A	0.9	0.3	1.0	0.5	0.5
FHA: MD (dB)	x	x	−0.69	−4.8	−11.74	x
Horizontal corneal diameter (mm)	7.5	9	9.5	9.5	^b^	^b^
Specular microscope (cells/m^2^)	^b^	^b^	1626	1636	^b^	^b^

*C/D* cup-to-disc ratio, *FHA* Humphrey Visual Field Analyzer, *MD* Mean Deviation, *NLP* No light perception; N/A: the patient can not finish the examination because of adherent leukoma; x: the patient can not finish the examination because of low vision; ^a^IOP after trabecylectomy; ^b^the patient refuses to do the examination; IOP reference range: ≤21 mmHg

**Fig. 2:**
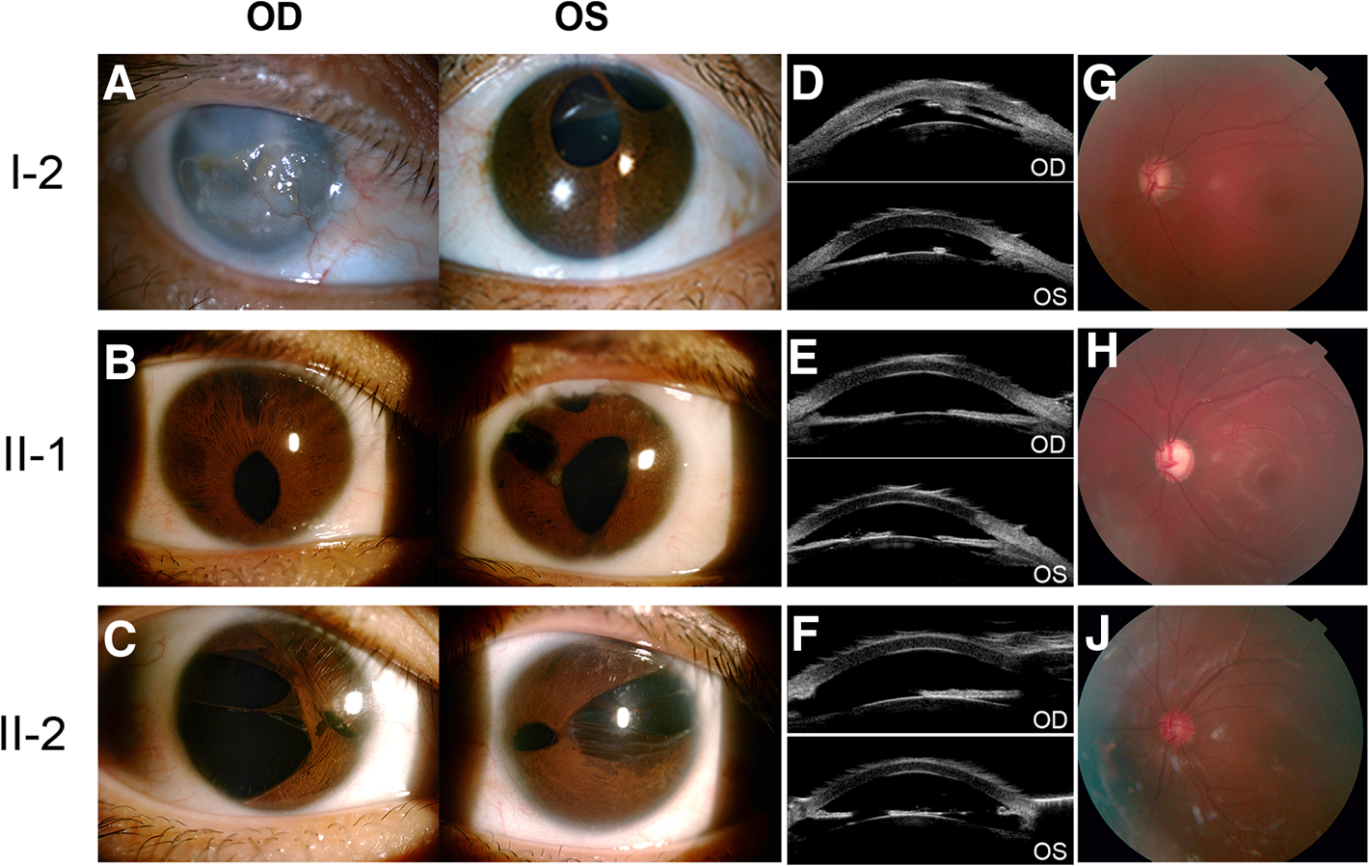
Ocular clinical features of affected family members with Axenfeld-Rieger syndrome. Anterior segment photography (**a**, **b** and **c**) showed different degrees of pupil deformation iris hypoplasia in both eyes and iris atrophy in both eyes for II-1 and II-2 and in the left eye for I-2. Adherent leukoma was observed in the right eye for I-2. Ultrasound biomicroscopy (**d**, **e** and **f**) demonstrated a shallow anterior chamber and different degrees of iris atrophy for I-2, II-1 and II-2. Fundus photography (**g**, **h** and **j**) of the left eye for the three affected members revealed abnormal CD ratio of 0.8 for I-2, 1.0 for II-1 and 0.5 for II-2

**Fig. 3:**
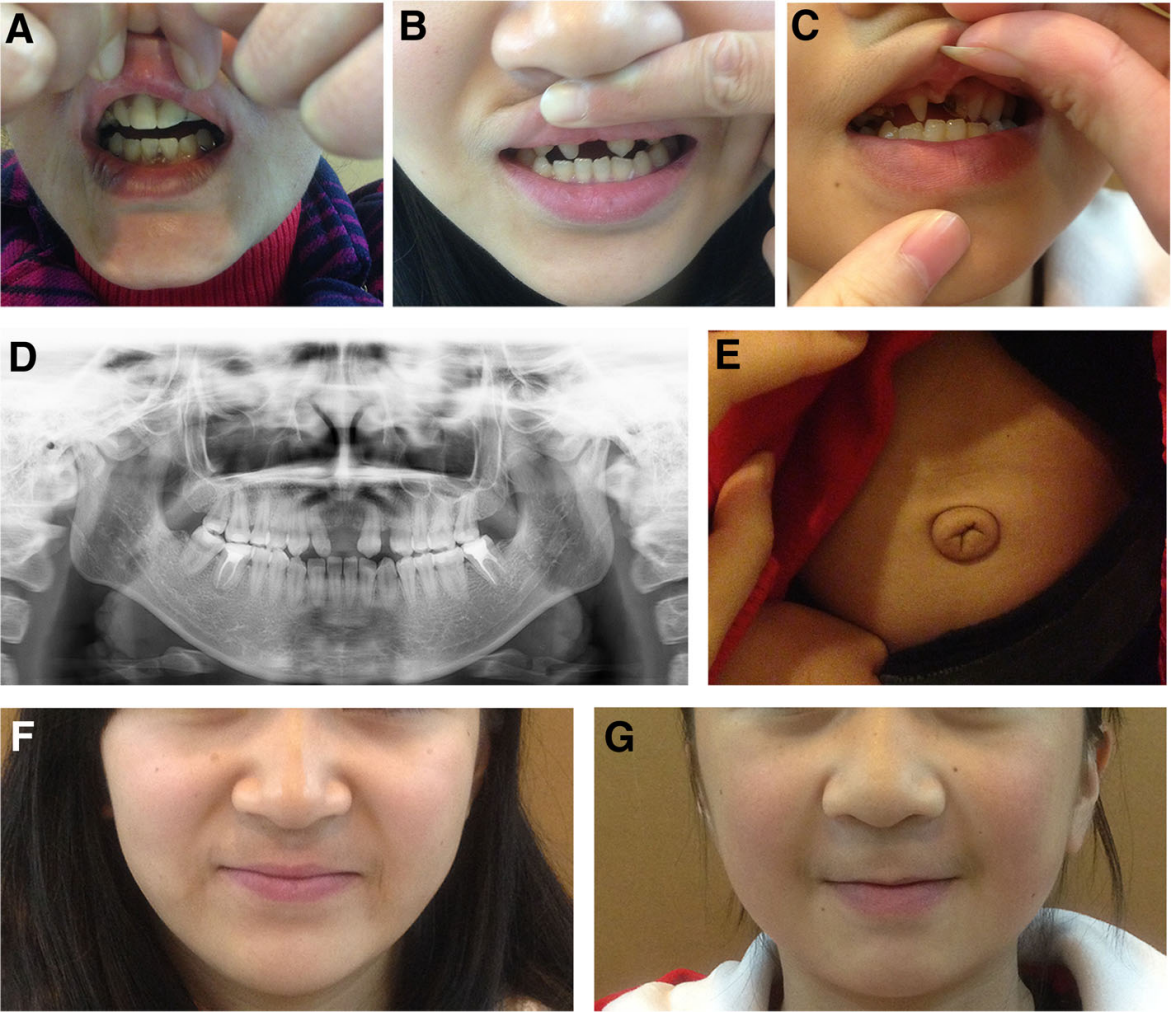
Systemic clinic features of affected family members with Axenfeld-Rieger syndrome. No microdontia was observed for I-2 because of a dental implant (**a**). Apparent microdontia for II-1 and II-2 (**b** and **c**). Dental panoramic radiography for II-1 (**d**), and II-2 had similar dental panoramic radiography. Umbilical defect (redundant periumbilical skin) for II-2 (**e**). All three patients exhibited the same sign when they underwent general physical examination. Broad nasal bridge of II-1 and II-2 (**f** and **g**)

All three cases showed that the abnormality of the anterior segment of their eyes included different degrees of pupil deformation, iris hypoplasia and iris atrophy in both eyes (except the mother since her right eye showed adherent leukoma). However, when we compared the degree of these symptoms, clearly II-2 was the most severe, followed by her elder sister (proband) with moderate anterior segment abnormality, and I-1 was shown to be the mildest compared to her daughters (Fig. 2 a, b and c). Slit-lamp examination revealed a shallow anterior chamber of both eyes in all cases, which was confirmed by UBM (Fig. 2 d, e and f). Apparent microdontia, broad nasal bridges and redundant periumbilical skin were observed in all patients (microdontia of the mother existed before the dental implant was performed 20 years ago) (Fig. 3).

The results of other ocular examinations, including BCVA, IOP, specular microscope, central corneal thickness (CCT), horizontal corneal diameter, Humphrey visual field analyzer (HFA) and the cup-to-disc (CD) ratio of each patients, are shown in Table 2 in details. After trabeculectomy for the left eye of the proband and regular medical therapy with Lumigan for her right eye, her IOP was controlled in an ideal range, which was ≤21 mmHg for the right eye and between 13 and 15 mmHg for the left eye. The BCVA of her left eye increased to 20/25.

When we compared the onset age (Table 1), II-2 was the earliest, with an age of 11 years, immediately followed by her elder sister (proband) with an age of 18 years. The onset age of I-1 was 29 years, which was considered to be the oldest age.

### Targeted exome sequencing and sanger validation

We selected proband II-1 for targeted exome sequencing analysis, which was designed to capture a 2637-bp DNA region in the two ARS candidate genes *PITX2* and *FOXC1* (Table 3). After sequencing, 82.03% of the qualified bases were mapped to the targeted sequence, with a mean read depth of approximately 151.61-fold, and the exon coverage rate was 96% (Table 3). After alignment and variation calling, 5 variations in the *PITX2* and *FOXC1* genes were identified, including 4 SNVs and 1 indel (data not shown). However, only the novel heterozygous frameshift variation c.515delA in *PITX2* was found in both the dbSNP database and 1000 genomes databases, which might lead to a truncated PITX2 protein (p.E172RfsX36).

**Table 3:** Summary of the targeted exome sequencing results

Sample	Targeted Gene	Targeted region (bp)	Targeted region coverage (%)	Targeted exon coverage (%)	Mean depth (×)	Mean depth > 30× (%)
II-1	FOXC1, PITX2	2637	82.03	96	151.61	63.48

By Sanger sequencing we validated that proband II-1 carries the heterozygous variation c.515delA in *PITX2* (Fig. 1d). Then, the exon was screened in other family members of the pedigree (I-2 and II-2), both of whom were patients, and they also carried heterozygous c.515delA variation (Fig. 1c, e). The variation completely cosegregated with the ARS phenotype among this family and was absent in the 100 unrelated controls (Fig. 1b). Taken together, our results indicated that the novel mutation c.515delA in *PITX2* is the disease-causing mutation in this ARS pedigree.

### *PITX2* expression analysis by real-time quantitative PCR

**Fig. 4:**
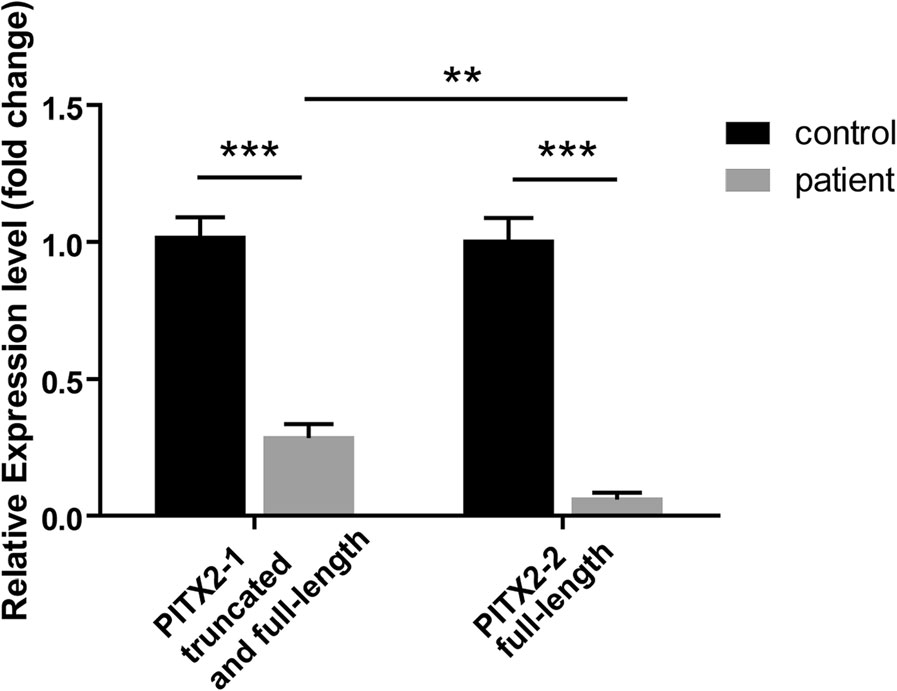
The mRNA expression of *PITX2* in lymphocytes of the affected individuals and the controls. The mean expression (±SEM) of *PITX2* mRNA in affected individuals (*n* = 3) and controls (*n* = 3) was measured by quantitative real-time PCR by primers before (PITX2–1) and after (PITX2–2) the mutant site. PITX2–1 columns represent both truncated and full-length pitx2 transcripts, while PITX2–2 columns indicate the full-length *PITX2* mRNA. **, *p* < 0.05; ***, *p* < 0.01

Because the frameshift mutation usually causes declined gene expression, we examined the expression of *PITX2* in peripheral blood lymphocytes of our three patients and three unrelated normal controls through real-time quantitative PCR. Two pairs of primers were designed according to the *PITX2* mRNA sequences before and after the mutant site c.515 to detect the mRNA expression of the pitx2 mRNA including truncated and full-length transcripts, or only full-length transcripts. As shown in Fig. 4, our patients revealed significantly decreased *PITX2* expression (29.19 ± 4.43%, *p* = 0.0008 for *pitx2*-primer 1 detection; 6.75 ± 1.73%, *p* = 0.0003 for *pitx2*-primer 2) compared with controls, indicating that the mutant would harm the expression of the *PITX2* gene. Moreover, full-length *PITX2* mRNA expression in patients (*pitx2*-primer 2 amplicon in patients) is much lower (*p* = 0.0091) than that of both truncated and full-length forms (primer 1 amplicon in patients) in patients, which means that the mutant DNA cannot normally express a full-length mRNA version but only a decreased truncated mRNA transcript. Taken together, these results suggested that *PITX2* c.515delA is a dominant negative mutation that both impaired the expression of *PITX2* and resulted in a truncated mRNA transcript.

## Discussion

In our present study, we identified a *PITX2* heterozygous frameshift mutation (c.515delA, p.E172RfsX36) that cosegregated with the ARS phenotype in our pedigree. Our real-time quantitative PCR results indicated a dominant negative effect of our mutation and decreased *PITX2* mRNA expression, which would be a pathogenic mechanism of our mutation.

ARS exhibits considerable phenotypic heterogeneity between affected individuals particularly with regard to the ocular anterior segment abnormalities, with or without other syndromic abnormalities. This can occur between different affected members within a single pedigree or between families carrying different mutations in the same gene [12]. In our pedigree, all three patients showed similar phenotypes, including anterior segment abnormality shown in Table 1, high IOP, increased C/D (cup-to-disc), microdontia, broad nasal bridge and redundant periumbilical skin. However, the level of severity for those symptoms and clinical characteristics differed from each other. Patient II-2 (the younger sister of the proband) was considered the most severe in the pedigree. The proband was more severe than their mother as well. The onset age of patient II-2 is younger than that of the proband. Thus, the mutation is at full penetrance, but disease severity shows variability.

*PITX2* encodes a 33 kDa bicoid-related homeodomain transcription factor, which belongs to the RIEG/PITX homeobox family. The protein contains a 60-amino-acid homeodomain at the N terminus and a 14-amino-acid C-terminal OAR domain, which mediate protein–protein interaction and self-inhibitory interaction with the N terminus [13]. The PITX2 protein participates in regulating the development of ocular anterior segment [14], as well as several nonocular tissues including branchial arches [15], heart [16], and the pituitary [17]. Mutations in *PITX2* have been associated with ARS and other anterior segment malformations, including Peter’s-like anomaly, iridogonio-dysgeniesis syndrome (IGDS) and iris hypoplasia (IH) [18–20]. The novel frameshift mutation c.515delA (p.E172RfsX36) leads to the adenine deletion in c.515 of *PITX2*, causing the glutamic acid in amino acid 172 replaced by arginin in the mutant transcript (Fig. 3b), which is predicted to create a shift in the reading frame and introduce a stop codon at position 208. The mutant amino acids are located in the highly conserved C-terminal domain of *PITX2*, which includes a transcriptional stimulatory domain in residues 160–232 and the transcriptional inhibitory OAR domain in residues 233–271 [21]. Moreover, the C-terminal region of PITX2 was suggested to facilitate intramolecular interactions that modulate DNA binding and transactivation [22]. Altogether, the protein-truncating mutation is predicted to disrupt C-terminal domain mediated protein interaction as well as its transcriptional regulation function. In addition, this mutation results in a significant and extreme decrease in the expression level of full-length PITX2 mRNA in affected individuals, which indicated that the mutant protein acts in a dominant negative manner, other than the haploinsufficient effect, to suppress wildtype *PITX2* expression. Because stringent control of PITX2 is required for normal ocular development and function, the alteration in the level of functional protein is the cause of the ARS phenotype in our pedigree.

## Conclusion

Our study described a novel *PITX2* frameshift mutation in the ARS family by combining exome sequencing with segregation analysis and expression analysis. A dominant negative effect leading to decreased protein expression as well as a truncated transcript caused by the mutation is the etiology of a Chinese family with Axenfeld-Rieger syndrome.

## Data Availability

The datasets used and/or analyzed during the current study are available from the corresponding author on reasonable request.

## Abbreviations

ARS: Axenfeld-Rieger syndrome
BCVA: Best-corrected visual acuity
C/D: Cup-to-disc
CCT: Central corneal thickness
ICE: Iridocorneal endothelial
IOP: Intraocular pressure
NCBI: National Center for Biotechnology Information
SNVs: Single nucleotide variations
UBM: Ultrasound biomicroscopy
